# Scutellarin Increases Cisplatin-Induced Apoptosis and Autophagy to Overcome Cisplatin Resistance in Non-small Cell Lung Cancer via ERK/p53 and c-met/AKT Signaling Pathways

**DOI:** 10.3389/fphar.2018.00092

**Published:** 2018-02-13

**Authors:** Chao-Yue Sun, Ying Zhu, Xiao-Feng Li, Xie-Qi Wang, Li-Peng Tang, Zu-Qing Su, Cai-Yun Li, Guang-Juan Zheng, Bing Feng

**Affiliations:** ^1^Guangdong Provincial Hospital of Chinese Medicine, The Second Clinical College of Guangzhou University of Chinese Medicine, Guangzhou University of Chinese Medicine, Guangzhou, China; ^2^Guangzhou Higher Education Mega Center, Clinical Medical College of Acupuncture and Rehabilitation, Guangzhou University of Chinese Medicine, Guangzhou, China

**Keywords:** scutellarin, cisplatin, NSCLC, apoptosis, autophagy

## Abstract

Cisplatin, as the first-line anti-tumor agent, is widely used for treatment of a variety of malignancies including non-small cell lung cancer (NSCLC). However, the acquired resistance has been a major obstacle for the clinical application. Scutellarin is a active flavone extracted from *Erigeron breviscapus Hand-Mazz* that has been shown to exhibit anticancer activities on various types of tumors. Here, we reported that scutellarin was capable of sensitizing A549/DDP cells to cisplatin by enhancing apoptosis and autophagy. Mechanistic analyses indicated that cisplatin-induced caspase-3-dependent apoptosis was elevated in the presence of scutellarin through activating extracellular signal-regulated kinases (ERK)-mediated p53 pathway. Furthermore, scutellarin also promoted cisplatin-induced cytotoxic autophagy, downregulated expression of p-AKT and c-met. Deficiency of c-met reduced p-AKT level, and inhibition of p-AKT or c-met improved autophagy in A549/DDP cells. Interestingly, loss of autophagy attenuated the synergism of this combination. *In vivo*, the co-treatment of cisplatin and scutellarin notably reduced the tumor size when compared with cisplatin treatment alone. Notably, scutellarin significantly reduced the toxicity generated by cisplatin in tumor-bearing mice. This study identifies the unique role of scutellarin in reversing cisplatin resistance through apoptosis and autophagy, and suggests that combined cisplatin and scutellarin might be a novel therapeutic strategy for patients with NSCLC.

## Introduction

Lung cancer is still the most prevalent malignant cancer worldwide with adverse clinical outcomes. Non-small cell lung cancer (NSCLC) accounts for 80–85% of lung cancer, with a low 5-years survival rate, approximately 13% (Siegel et al., 2016). Cisplatin is first-line anti-tumor drug for the treatment of advanced NSCLC (Miller et al., 2016). However, due to the frequent occurrence of acquired resistance, the use of cisplatin is limited (Galluzzi et al., 2012). Also, the molecular mechanisms by which cisplatin leads to resistance is not fully clear. Therefore, investigating the underlying mechanism involved in cisplatin resistance, and searching for an effective component that can reverse the resistance are clinically necessary.

Apoptosis is a form of cell death, and as such it has been frequently induced by chemotherapy agents (Zhu et al., 2017). Autophagy, also called “self-eating,” is an evolutionarily conserved catabolic process that sequesters unnecessary proteins and organelles to keep cell homeostasis (Yang and Klionsky, 2010). It is well studied that autophagy functions as a key factor in the tumorigenesis and cancer chemotherapy (Li Y.J. et al., 2017). To date, however, there are still a troublesome question: autophagy is a friend or foe for cancer treatment? Recently, accumulating evidence has been demonstrated that autophagy protects cancer cells when treated by chemotherapy, resulting in drug resistance (Wang et al., 2013). Accordingly, autophagy inhibitors are often recognized as a potential therapeutic strategy to enhance the cytotoxicity of chemotherapeutic drug (McAfee et al., 2012; Jin et al., 2017). However, unlike above reports, drug-induced autophagy can sensitize cancer cells to chemotherapy (Li and Zhang, 2017). Thus, the role of autophagy in cancer therapy remains not completely understood.

Extracellular signal-regulated kinases (ERK) plays a crucial role in the regulation of tumorigenesis, cell proliferation and cancer chemotherapy (Fremin and Meloche, 2010). Paradoxically, overexpression of ERK in advanced lung adenocarcinoma, is associated with worse chemotherapeutic outcome and a poor prognosis (Tsujino et al., 2016). However, on the other hand, ERK can be activated by many anti-cancer drugs, such as cucurbitacin to induce high autophagy, resulting tumor cell death (Wu et al., 2016). Thus, it is of paramount importance to explore the role of ERK pathway in cancer chemotherapy.

The c-met signaling is hyperactivated in a number of malignancies including NSCLC, and has been implicated in cancer initiation, progression and therapeutic resistance (Gozdzik-Spychalska et al., 2014). Recent study has demonstrated that cisplatin resistance cells A549/DDP exerted up-regulation of c-met, and suppression of the c-met by salvianolic acid A could reverse cisplatin resistance (Tang et al., 2017). Notably, the c-met signaling has an important role in the regulation of autophagy (Akl et al., 2015). For example, in lung adenocarcinoma, loss of c-met contributed to the induction of autophagy (Liu et al., 2013). Unfortunately, in cisplatin resistance, the role of c-met in regulating autophagy remains unresolved.

Scutellarin (4′,5,6-trihydroxyflavone-7-*O*-glucuronide), is a active flavone extracted from Chinese traditional herb *Erigeron breviscapus Hand-Mazz*, which was generally used for treatment of cerebral vascular diseases (Wang et al., 2017). Recently, scutellarin has been shown to exhibit anticancer activities on various types of tumors, such as hepatocellular carcinoma, colorectal cancer and tongue squamous carcinoma (Li et al., 2013; Ke et al., 2017; Yang et al., 2017), although the underlying mechanisms have not been fully determined. However, little is known about whether scutellarin can potentiate antitumor activity of cisplatin in NSCLC. In this study, we aimed to explore the mechanisms underlying synergistic interactions between scutellarin and cisplatin, and provided a new therapeutic target for reversing cisplatin resistance.

## Materials and Methods

### Reagents and Antibodies

Scutellarin (purity ≥ 98%) was obtained from Sigma–Aldrich (St. Louis, MO, United States), and dissolved in PBS (PH 7.4). Cisplatin was provided by Yangtze River Pharmaceutica, and diluted in culture medium. U0126, hydroxychloroquine (HCQ), MK-2206, pifithrin-μ (PFT) and crizotinib were purchased from Selleckchem (Houston, TX, United States). Primary antibodies against β-actin (catalog number: 4970S), LC3 (catalog number: 12741S), p62 (catalog number: 39749S), p53 (catalog number: 2527S), caspase-3 (catalog number: 9662S), cleavaged caspase-3 (catalog number: 9664S), PARP (catalog number: 9454S), ERK1/2 (catalog number: 4695S), p-ERK1/2 (catalog number: 8544S), c-met (catalog number: 4560S), AKT (catalog number: 2920S), P-AKT (catalog number: 4060P), together with secondary HRP-conjugated goat anti-rabbit antibody were purchased from Cell Signaling Technology (Danvers, MA, United States).

### Cell Culture

The human lung adenocarcinoma cell lines A549, PC-9, H1975, and A549/DDP were obtained from the Chinese Academy of Sciences (Shanghai, China). A549/DDP cell line was generated from its parental A549 by step-dose selection *in vitro*. A549 cells were exposed to increasing concentrations ranging from 1 to 8 μM for 4 months. After establishment, the resistant index of the A549/DDP at 48 h was 37.37. Cells were maintained in RPMI 1640 supplemented with 10% fetal bovine serum (FBS; Gibco Laboratories, Grand Island, NY, United States). In addition, the culture medium for A549/DDP cells contained 2 mg/L cisplatin to keep its drug-resistant phenotype.

### MTT Assay and Synergy Analyses

To validate chemosensitivity of A549/DDP cells to cisplatin, the MTT assay was firstly performed. In summary, cells were seed into 96-well plates (5 × 10^3^ cells/well), and cultured overnight for adherence. Cells were exposed to different concentrations of cisplatin (0.625, 1.25, 2.5, 5, 10, and 20 μg/ml), scutellarin (10, 20, 40, 80, 120, and 160 μM), or their combination. After treatment for 24 or 48 h, 20 μL MTT (5 mg/ml) was added to each well and incubated for another 4 h. Subsequently, the medium was discarded and replaced with 150 μL DMSO. The absorbance at 490 nm wavelength was measured using a multiwell spectrophotometer (Bio-Rad, Hercules, CA, United States). To calculate the combination index (CI) of cisplatin and scutellarin, CalcuSyn software package (version 2.1) was used (Omar et al., 2016).

### Flow Cytometry

For apoptosis studies, cells were treated with cisplatin, or scutellarin, or in combination for 48 h. Following the cell collection after being centrifuged, cells were subsequently re-suspended with 500 μL binding buffer. Cells were stained with 5 μL Annexin V-FITC and 10 μL PI solution provided in an Annexin V-FITC apoptosis detection kit (MultiSciences Biotech Co., Ltd.). The apoptotic cells were detected by a flow cytometer (BD Biosciences, San Jose, CA, United States).

### Western Blot Analysis

Total protein was harvested using standard RIPA buffer that contained 1% protease and phosphatase inhibitors (Thermo Fisher Scientific, Canoga Park, CA, United States). Equal proteins were subjected to electrophoresis on a 8 or 12% SDS-polyacrylamide gel, then transferred into PVDF membrane. After blocked in 5% skim milk for an hour, the membrane was incubated in primary antibodies overnight at 4°C. Then, the membrane were hybridized with the secondary horseradish peroxidase-conjugated antibodies at room temperature for an hour. Finally, protein bands were visualized by the enhanced chemiluminescence (ECL) system (Millipore, United States), and the expression of protein was measured using the ImageJ software. Here, β-actin was used as an internal control.

### Transmission Electron Microscopy (TEM)

Cells were collected and washed by PBS, and promptly immersed in a fixative solution of 2.5% glutaraldehyde for 4 h at 4°C. The samples were postfixed in 1.5% osmium tetroxide, and then dehydrated in a graded series of ethanol. Ultrathin sections (50 nm) were cut, followed by dyed with 2.5% uranyl acetate and 1% lead citrate. The samples were examined with a electron microscope (Hitachi H-7650, Tokyo, Japan) at 80 kV, and the images were captured using a Veleta TEM camera.

### Establishment of A549/DDP-Luc Cells

For establishment of A549/DDP-Luciferase (A549/DDP-Luc) cells, a Hind-III and Xba-I fragment of the luciferase was prepared for pGL4.13-Promoter (Promega, Fitchburg, WI, United States) and inserted into the PRC/CMV_2_ vectors. The resulting PRC/CMV_2_-Luc recombinant plasmid was transfected into A549/DDP cells to establish A549/DDP-Luc, followed by G418 screening to obtain unicellular resistant clones. The clone cells stably expressing strong luciferase were selected for animal experiment by luciferase activity test.

### *In Vivo* Xenograft and Treatment Experiments

The animal procedures were approved by the Animal Care and Use Committee of Guangdong Provincial Hospital of Chinese Medicine (the Ethics Approval Number 2016023) and the Declaration of the National Institutes of Health Guide for Care and Use of Laboratory Animals. A549/DDP-Luc cells (4 × 10^6^) were subcutaneously injected into the right flank of 4-to-6 week-old female BALB/c nude mice, which were purchased from Guangdong Medical Laboratory Animal Center (Fushan, Guangdong, China) when the tumor reached approximately 100 mm^3^, mice were randomly divided into four groups (*n* = 8 each): the vehicle; the cisplatin alone; the scutellarin alone; and cisplatin + scutellarin. Cisplatin (5 mg/kg) were intraperitoneally given every 3 days, while scutellarin (60 mg/kg) were orally administered daily. Cisplatin was diluted using normal saline for the certain dosage, and scutellarin was dissolved in PBS (PH 7.4). The tumor dimensions and body weight were measured per 3 days, and tumor volume was calculated as follows: Volume = (Length × width^2^) × 0.5. After treatment for 21 days, mice were humanely euthanized, and the tumor tissues were subsequently harvested for further analysis.

### Statistical Analysis

Data are depicted as the mean ± SEM. One-way analysis of variance (ANOVA) was used for multiple comparisons among three or more groups, while sample *t*-test was utilized for comparisons between two groups. *p* < 0.05 was interpreted to indicate statistical significance.

## Results

### Scutellarin Enhanced the Drug Susceptibility of Cisplatin in A549/DDP Cells

**FIGURE 1:**
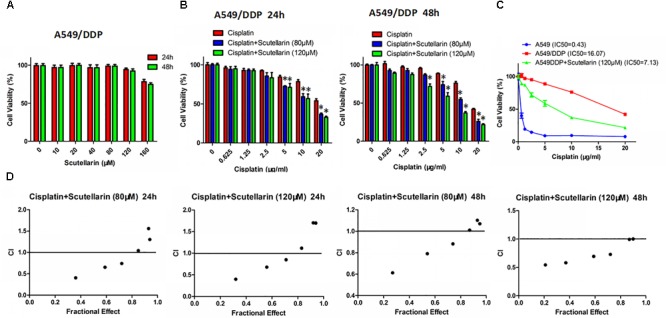
Scutellarin enhanced the drug susceptibility of cisplatin in A549/DDP cell. **(A)** A549/DDP cells were treated with different concentrations of scutellarin for 24 or 48 h, the cell viability was determined by the MTT assay. **(B)** Cells were treated with cisplatin combined with scutellarin (80 or 120 μM), cell viability was measured by the MTT assay. **(C)** The sensitivity of A549 and A549/DDP to cisplatin was measured by MTT assay, and 120 μM scutellarin reduced IC50 of cisplatin in A549/DDP cells. **(D)** Drug synergism are expressed as fraction affected (Fa) curves and combination index (CI) plots. CI as a indicator of synergistic effects of cisplatin and scutellarin (additive effect, CI = 0.9–1.1; slight synergism, CI = 0.7–0.9; synergism, CI = 0.3–0.7; strong synergism, CI = 0.1–0.3). ^∗^*p* < 0.05.

Previously, we found that scutellarin potently suppressed the cell viability of NSCLC parental cells including A549, PC-9, H1975 (**Supplementary Figure S1B**), whereas the cytotoxic effect of scutellarin on cisplatin-resistant A549/DDP cells was dismal (**Figure 1A**). However, co-treatment of scutellarin and cisplatin significantly sensitized A549/DDP cells to cisplatin (**Figure 1B**). Here, we compared cisplatin-resistant cells A549/DDP with the parental A549 cells, A549/DDP showed high resistance to the DDP challenge. The IC50 of A549 and A549/DDP cells was 0.43 and 16.07 μg/ml, respectively, and the resistant index was 37.37 (**Figure 1C**). A CI was used to assess synergistic effects of cisplatin with scutellarin. Combinated cisplatin and scutellarin at 80, 120 μM showed a abvious synergism (**Figure 1D**). Thus, cisplatin and scutellarin yield a synergistic effect in killing A549/DDP cells. Specifically, 120 μM scutellarin did not yield measurable impact on cell viability of A549/DDP cells, but clearly enhanced the sensitivity of A549/DDP to cisplatin. Also, as shown in **Figure 1C**, the efficiency of 10 μg/ml cisplatin combined with 120 μM scutellarin peaked at 48 h. Of note, 120 μM scutellarin obviously reduced the IC50 of cisplatin in A549/DDP cells. Thus, 10 μg/ml cisplatin and 120 μM scutellarin were used for further study.

### Scutellarin Enhanced Cisplatin-Induced p53-Dependent Apoptosis

**FIGURE 2:**
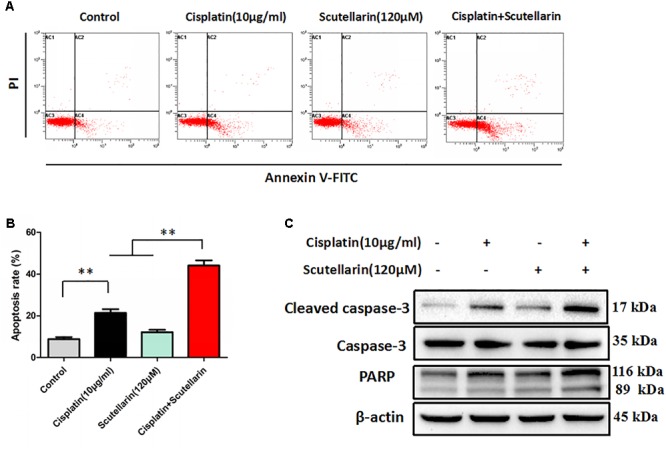
Scutellarin enhanced cisplatin-induced apoptosis. **(A)** Cells were exposed to cisplatin with or without scutellarin for 48 h, cell apoptosis was measured by flow cytometric analysis. **(B)** Apoptosis rate in each group. **(C)** Western blot analysis showing caspase-3, cleaved caspase-3 and PARP expression levels in A549/DDP cells treated as indicated. Actin was used as loading control. Data are representative of three independent experiments (mean ± SEM). ^∗∗^*p* < 0.01.

We next examined whether scutellarin could enhance cisplatin-induced apoptosis using flow cytometry. Scutellarin increased cisplatin-induced apoptosis by greater than 24%, when compared with cisplatin alone (**Figures 2A,B**). Caspase-3 is cleaved and activated during apoptosis, and in turn, caspase-3 cleaves PARP (Xu et al., 2016). In addition to flow cytometry, we tested the expression of caspase-3, cleavage of caspase-3 and PARP. Combination of cisplatin and scutellarin significantly increased cleavage of caspase-3 and PARP when compared with cisplatin alone (**Figure 2C** and **Supplementary Figure S2A**).

**FIGURE 3:**
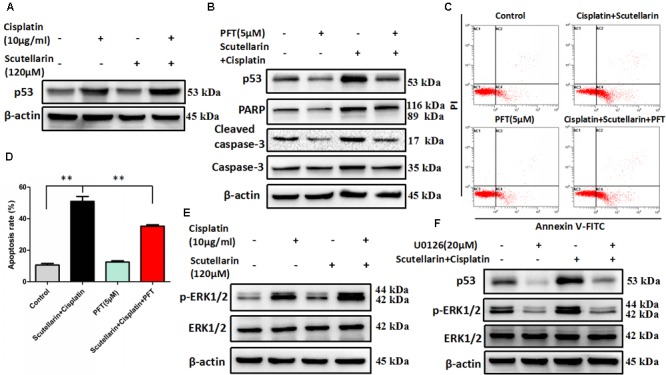
Scutellarin enhanced cisplatin-induced apoptosis through the ERK1/2-mediated p53 signaling pathways. **(A)** Western blot analysis of p53 protein in A549/DDP cells treated by cisplatin, or scutellarin, or the combination. **(B)** The effect of p53 inhibitor PFT on p53, caspase-3, cleaved caspase-3 and PARP expression levels was measured by western blot analysis. **(C,D)** Flow cytometric analysis showing apoptosis in cells treated by cisplatin plus scutellarin, or PFT, or the combination. **(E)** The expression of p-ERK1/2, ERK1/2 were determined by western blot analysis. **(F)** The level of p-ERK1/2, ERK1/2, p53 in A549/DDP cells were measured in the presence of ERK1/2 specific inhibitor U0126. Data are representative of three independent experiments (mean ± SEM). ^∗∗^*p* < 0.01.

Given that p53 plays a distinct role in cisplatin-mediated apoptosis in cancer cells (Li et al., 2015), we examined p53 expression by western blots. In this study, cisplatin-resistant A549/DDP cells still retain the wild-type p53. In the presence of scutellarin, the cisplatin-induced expression of p53 was markedly enhanced (**Figure 3A** and **Supplementary Figure S3A**). To identify whether the heightened apoptotic response was p53-dependent, we next treated A549/DDP cells with a specific p53 inhibitor, pifithrin-μ (PFT). As expected, inhibition of p53 by PFT blocked cisplatin plus scutellarin-induced cleavage of caspase-3 and PARP (**Figure 3B** and **Supplementary Figure S3B**). Moreover, PFT markedly reduced cell apoptosis induced by co-treatment of cisplatin and scutellarin (**Figures 3C,D**). Thus, scutellarin enhanced cisplatin-induced apoptosis through activation of p53 signaling pathway.

### Scutellarin Enhanced Cisplatin-Induced p53-Dependent Apoptosis through Activating the ERK1/2 Signaling Pathway

It is well known that ERK pathway has been reported to regulate p53-dependent apoptosis (Lv et al., 2013). In line with this, we found that p-ERK1/2 level showed a very obvious increase in response to cisplatin plus scutellarin treatment in A549/DDP cells (**Figure 3E** and **Supplementary Figure S3C**). Similarly, we used a ERK inhibitor, U0126 to determine whether ERK pathway participated in scutellarin and cisplatin-induced apoptosis. Western blot analysis revealed that U0126 reversed the high expression of p53 induced by the combination treatment in A549/DDP cells (**Figure 3F** and **Supplementary Figure S3D**). Thus, scutellarin enhanced cisplatin-induced p53-dependent apoptosis through activating the ERK1/2 signaling pathway.

### Scutellarin Increased Cisplatin-Induced Autophagy

**FIGURE 4:**
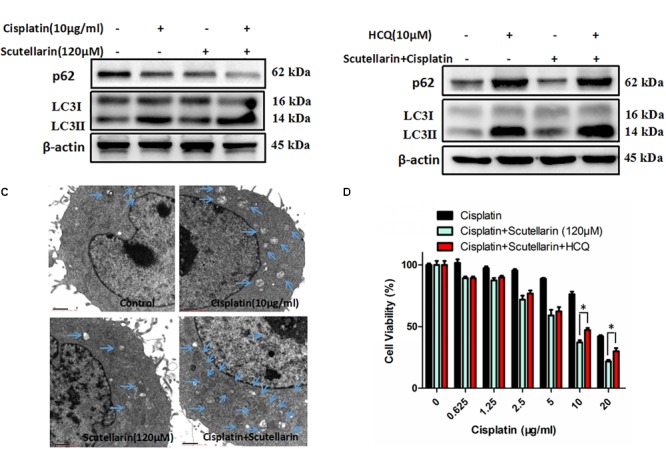
Scutellarin increased cisplatin-induced autophagy. **(A)** A549/DDP cells were treated with the indicated concentrations of cisplatin or scutellarin, or the combination for 48 h, the expression of p62 and conversion of LC3-I to LC3-II were measured by western blotting. **(B)** Western blot analysis of p62 and LC3 in A549/DDP cells incubated with autophagy inhibitor HCQ. **(C)** Quantification of autophagic vacuoles in A549/DDP cells was analyzed by TEM. **(D)** The effect of HCQ on sensitivity of cisplatin and scutellarin was detected by MTT assay. Data are representative of three independent experiments (mean ± SEM). ^∗^*p* < 0.05, ^∗∗^*p* < 0.01.

Autophagy plays a central role in governing chemotherapy resistance, yet its involved mechanisms are largely unknown. Here, we tested autophagy-related proteins, LC3 and p62, using western blot analysis. In the presence of scutellarin, the level of LC3-II was greatly increased in A549/DDP cells treated with cisplatin, whereas p62 was degraded (**Figure 4A** and **Supplementary Figure S4A**). Meanwhile, we employed TEM to verify the autophagy induced by cisplatin, or scutellarin, or in combination. As observed, the number of autophagic vacuoles was elevated in A549/DDP cells exposed to co-treatment of cisplatin and scutellarin, compared with cisplatin alone, or scutellarin alone (**Figure 4C**), indicating scutellarin indeed enhanced cisplatin-induced autophagy. In addition, autophagy inhibition achieved by using autophagy inhibitor HCQ, dramatically impaired autophagic flux (**Figure 4B** and **Supplementary Figure S4B**). Interestingly, MTT assay results revealed that HCQ treatment slightly contributed to cisplatin resistance in A549/DDP cells (**Figure 4D**). Together, these findings suggested that scutellarin was able to promote cisplatin-induced autophagy, of which was not cytoprotective, but lead to cell death.

### Scutellarin Increased Cisplatin-Induced Autophagy through Suppressing c-met/AKT Signaling Pathway

**FIGURE 5:**
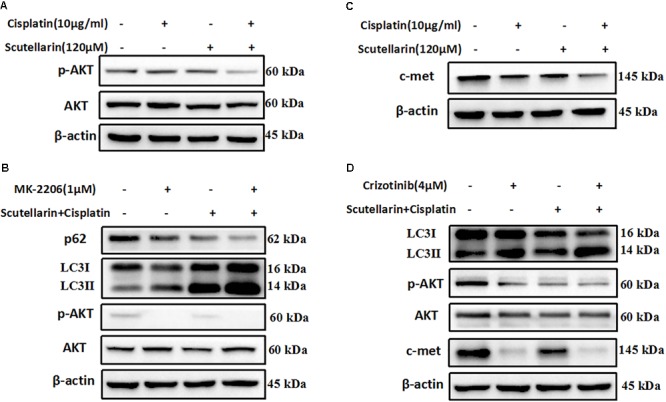
Scutellarin increased cisplatin-induced autophagy through suppressing c-met/AKT signaling pathways. **(A)** Cells were treated by cisplatin with or without scutellarin, the protein expressions of AKT and p-AKT were determined by western blotting. **(B)** The expression levels of LC3, AKT, p-AKT were detected by western blotting in A549/DDP cells coincubated with MK-2206. **(C)** Western blot analysis of the protein of c-met. **(D)** The effect of crizotinib on the expression of LC3, AKT, p-AKT, c-met was analyzed by western blot analysis.

Previous researches have been shown that c-met and AKT both play an important role in regulating autophagy (Heras-Sandoval et al., 2014; Li N. et al., 2017). Here, we also found that the expression of c-met and p-AKT was higher in cisplatin-resistant A549/DDP than A549 cells (**Supplementary Figure S1A**). In addition, hence, we question whether the increased autophagy was associated with above pathways, and we profiled AKT and c-met using western blot analysis. Comparatively, combined treatment with cisplatin and scutellarin reduced the expression of p-AKT, whereas the AKT level showed no significant difference (**Figure 5A** and **Supplementary Figure S5A**). In addition, inhibition of AKT by pharmacological inhibitor MK-2206 decreased p-AKT, but significantly enhanced autophagy marker expression LC3-II, and attenuated the level of p62 (**Figure 5B** and **Supplementary Figure S5B**). Moreover, in the presence of scutellarin, cisplatin caused significant suppression of c-met in A549/DDP cells (**Figure 5C** and **Supplementary Figure S5C**). Crizotinib is a potent and specific small-molecule inhibitor of c-met that binds to the hinge region of c-met in a bidentate manner and competes with ATP binding in c-met (Gandhi and Jänne, 2012). Here, the addition of c-met inhibitor, crizotinib abolished c-met expression, impaired p-AKT, and promoted high autophagy (**Figure 5D** and **Supplementary Figure S5D**). Taken together, these data showed that scutellarin enhanced cisplatin-induced autophagy by suppressing c-met/AKT pathways.

### Scutellarin Enhanced Antitumor Efficacy of Cisplatin *in Vivo*

**FIGURE 6:**
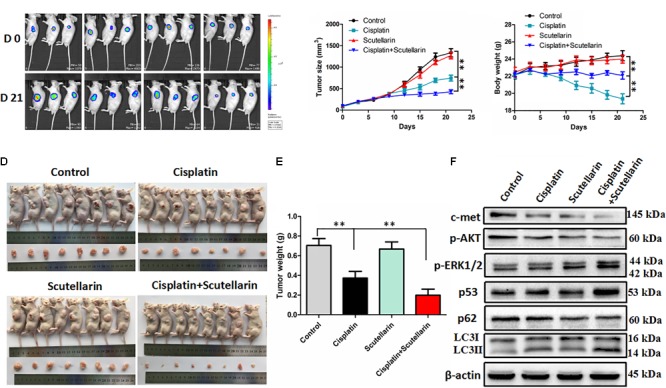
Scutellarin enhanced antitumor efficacy of cisplatin *in vivo*. The A549/DDP-Luc cells were subcutaneously implanted BALB/c nude mice, and the mice were treated with cisplatin with or without scutellarin. **(A)** Tumor growth was monitored by IVIS, representative bioluminescence images of tumor in mice are shown. **(B)** The tumor dimensions were measured per 3 days using a digital caliper, tumor size in each group is shown. **(C)** Body weight of each group (*n* = 8). **(D)** After treatment for 21 days, mice were humanely euthanized, and the tumor tissues were subsequently harvested. **(E)** Tumor weight of each group (*n* = 8). **(F)** The expression levels of LC3, p62, p53, p-ERK1/2, p-AKT, c-met in mice tumor were detected by western blot analysis. Data are representative of three independent experiments (mean ± SEM). ^∗∗^*p* < 0.01.

To verify our previous conclusions, we established xenograft mouse model. The A549/DDP-Luc cells were subcutaneously implanted into BALB/c nude mice, and the mice were treated with cisplatin with or without scutellarin. As shown in **Figures 6A,B,D,E**, cisplatin significantly inhibited tumor growth, and this efficiency was significantly enhanced by scutellarin treatment, whereas the anti-tumor activity of scutellarin alone was dismal. Furthermore, due to the toxicity of cisplatin, the body weight of mice treated by cisplatin was decreased. However, in the presence of scutellarin, the weight reduction was recovered (**Figure 6C**). Moreover, the combination of cisplatin with scutellarin increased the expression of LC3-II, p53, p-ERK1/2, and reduced p-AKT and c-met, compared with cisplatin alone (**Figure 6F** and **Supplementary Figure S6A**). Collectively, scutellarin could notably improve anti-tumor effect of cisplatin, and reduce the toxicity generated by cisplatin *in vivo*.

## Discussion

Cisplatin, one of the most commonly chemotherapeutic agent, has been widely used to treat different types of cancer including NSCLC (Kim et al., 2017). However, its tumoricidal efficacy is often limited due to the rapid emergence of acquired resistance. Multiple mechanisms contributed to cisplatin resistance have been documented, such as decreased drug absorption, DNA repair, inactivated apoptosis programs (Fang et al., 2017). Unfortunately, there remains a lack of targeted treatment strategies focusing on reversing cisplatin resistance. To circumvent this difficulty, we focused on looking for novel drugs to suppress chemotherapy resistance. Our results offer the first evidence, to our knowledge, that scutellarin improved the sensitivity of cisplatin-resistant cells A549/DDP to cisplatin. In addition to A549/DDP cells, our study shows that scutellarin could potentiate antitumor activity of cisplatin in NSCLC line PC-9, suggesting the synergism of scutellarin and cisplatin was applied to NSCLC cells (**Supplementary Figure S1C**).

Apoptosis is the predominant manner of cisplatin-induced cell death. However, cancer cells can develop its own specific strategies to evade apoptosis, which facilitates their survival and promote resistance to anticancer therapies (Ravegnini et al., 2017). Caspase-3 is a apoptotic effector that has been well studied to be involved in cisplatin-induced apoptosis (Shen et al., 2007). In this study, cisplatin markedly induced caspase-3-dependent apoptosis, of note, its efficiency was enhanced by scutellarin. Accumulating evidence has shown that p53 serves as a key tumor suppressor, which is essential for induction of cisplatin-induced apoptosis (Yang et al., 2012). It has been proposed that the presence of functional p53 facilitates the cytotoxic effects of chemotherapeutic drugs including cisplatin (Herudkova et al., 2017). For instance, in head and neck squamous cell carcinoma, small molecules targeted p53-reactivation have been shown to induce apoptosis and enhance chemotherapeutic cytotoxicity (Roh et al., 2011). Here, we demonstrated that scutellarin increased cisplatin-induced p53 activation, resulting in apoptotic cell death. This is especially important given that p53 activation is mainly responsible for enhancement of cisplatin-induced apoptosis by scutellarin.

Extracellular signal-regulated kinases, an prototypic MAPK (mitogen-activated protein kinase), is involved in Ras/Raf/ERK pathway that is hyperactivated in a high many types of tumors, which drives cancer growth, inhibits apoptosis, promotes angiogenesis and invasion (Miyake et al., 2015). As such, small-molecule inhibitors targeting ERK signaling, such as RAF or MEK inhibitors, have shown to promise as efficient cancer therapeutics (Samatar and Poulikakos, 2014). However, ERK pathway also can be activated by antitumor agents to trigger its downstream tumor suppressor proteins (Lv et al., 2013). Meanwhile, cisplatin-induced ERK activation is an key regulator of the p53 response to DNA damage caused by cisplatin (Persons et al., 2000). Our data show that scutellarin serves an ERK inducer that potentiated cisplatin-induced ERK activation. Inhibition of ERK by U0126 suppressed p53 expression. Thus, co-treatment of cisplatin and scutellarin induced p53-mediated apoptosis via activating ERK pathway.

Autophagy or “self-eating,” has been vividly regarded as janus-faced player due to its dual function in cancer progression (Gugnoni et al., 2016). The role of autophagy in cancer, however, is still paradoxical. On the one hand, autophagy plays a central role in suppressing tumor initiation by inhibiting necrosis and immune cell infiltration during tumorigenesis (Eritja et al., 2017). However, on the other side, autophagy can confer cancer cells the ability to protect itself in response to new hostile environments, such as hypoxic and nutrient-deficient (Zhan et al., 2016). Notably, some tumor cells are able to hijack the adaptation cues to cope with autophagy triggered by anti-tumor therapy (Kondo et al., 2005). In certain condition, autophagy inhibition can enhance the efficacy of anticancer treatments. Thus, the role of autophagy in cancer therapy has not been clear. During autophagy activation, lots of double-membrane electron-dense autophagosomes that capture unnecessary contents fuse with lysosomes or vacuoles to form autolysosomes, where these dysfunctional organelles are degraded (Li Y. et al., 2017). Meanwhile, in particular, accumulation of LC3-II and p62 degradation are regarded as the induction of autophagy (Guo et al., 2013). Western blots and TEM results demonstrated combination of cisplatin and scutellarin markedly increased the induction of autophagy. Interestingly, deficiency of autophagy slightly contributed to cisplatin resistance in A549/DDP cells. These observations elucidated that scutellarin was able to promote cisplatin-induced autophagy, of which was not cytoprotective, but lead to cell death.

It is well established that PI3K/AKT/mTOR pathway is emerging as important player in the regulation of autophagy in various types of cancers (Chen et al., 2017). For example, in prostate cancer, inhibition of the PI3K/AKT/mTOR pathway is sufficient to activate autophagy (Butler et al., 2017). Indeed, co-treatment of cisplatin and scutellarin significantly decreased the level of phosphorylated AKT in A549/DDP cells, and the AKT inhibitor MK-2206 enhanced the induction of autophagy. The c-met oncogene has been identified as a potential target for cancer therapy. Activation of c-met contributes to the proliferation and invasion of cancer cells, and has been also considered as a biomarker in several tumor types (Blumenschein et al., 2012). Of note, AKT is regarded as a downstream factor of the c-met pathway (Peters and Adjei, 2012). Here, we found that cisplatin or scutellarin alone could suppressed c-met level, however, the efficiency of combination treatment was stronger. Inhibition of c-met by crizotinib attenuated p-AKT level, but induced high autophagy. These findings clearly indicated that scutellarin enhanced cisplatin-induced autophagy via suppressing the c-met/AKT signaling pathway. Furthermore, our *in vivo* experiment indicated that scutellarin significantly enhanced cisplatin-suppressed tumor growth, and reduced the toxicity generated by cisplatin.

**FIGURE 7:**
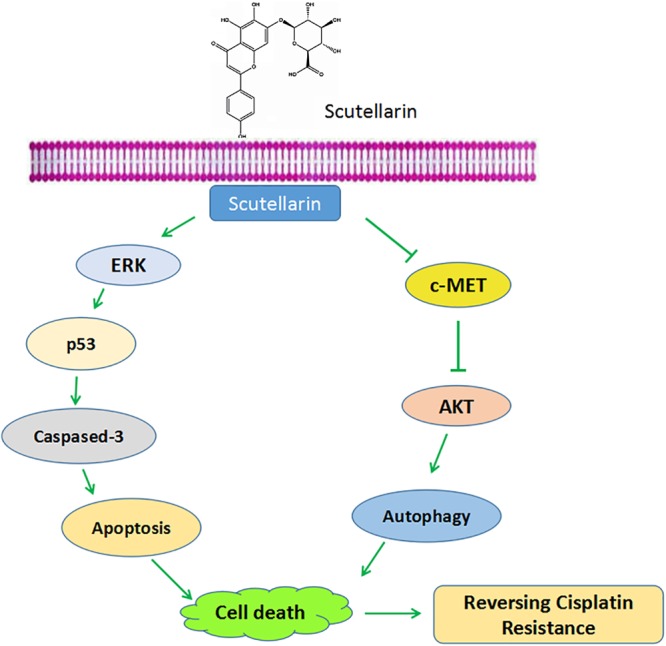
Schematic diagram shows scutellarin increases the sensitivity of non-small cell lung cancer to cisplatin via ERK/p53 and c-met/AKT signaling pathways.

In sum, our results showed that scutellarin reversed cisplatin resistance in NSCLC cells. Mechanically, scutellarin enhanced cisplatin-induced apoptosis and autophagy via ERK/p53 or c-met/AKT signaling pathways, suggesting that ERK and c-met signalings were the direct target of scutellarin (**Figure 7**). Notably, scutellarin can reduce the toxicity associated with repeated administration of cisplatin in tumor-bearing mice. These findings suggest that the combination of cisplatin treatment with scutellarin can be effectively applied for the treatment of NSCLC. In this study, A549/DDP cells still retain the wild-type p53, it is not clear whether the combination therapy is effective to p53-mutant lung cells. Thus, further experiments should performed to demonstrate whether scutellarin can enhance the sensitivity of p53-mutant cells to cisplatin.

## Author Contributions

C-YS designed the experiments and drafted the manuscript. YZ and X-FL revised the manuscript. X-QW and L-PT carried out western blots and statistical analyses. Z-QS and C-YL performed the animal experiments. G-JZ and BF supervised the study.

## Conflict of Interest Statement

The authors declare that the research was conducted in the absence of any commercial or financial relationships that could be construed as a potential conflict of interest.

## Supplementary Material

FIGURE S1 **(A)** The expression levels of c-met, p-AKT in A549 and A549/DDP cells were detected by western blot analysis. **(B)** PC-9 cells were treated with different concentrations of scutellarin, cell viability was measured by the MTT assay. **(C)** PC-9 cells were treated with cisplatin combined with scutellarin (40 μM), cell viability was measured by the MTT assay. ^∗^*p* < 0.05.

FIGURE S2 **(A)** Western blot analysis showing caspase-3, cleaved caspase-3 and PARP expression levels in A549/DDP cells treated with cisplatin, or scutellarin, or the combination. Actin was used as loading control.

FIGURE S3 **(A)** Western blot analysis of p53 protein in A549/DDP cells treated by cisplatin, or scutellarin, or the combination. **(B)** The effect of p53 inhibitor PFT on p53, caspase-3, cleaved caspase-3 and PARP expression levels were measured by western blot analysis. **(C)** The expression of p-ERK1/2, ERK1/2 were determined by western blot analysis. **(D)** The level of p-ERK1/2, ERK1/2, p53 in A549/DDP cells were measured in the presence of ERK1/2 specific inhibitor U0126.

FIGURE S4 **(A)** A549/DDP cells were treated with cisplatin or scutellarin, or the combination for 48 h, the expression of p62 and conversion of LC3-I to LC3-II were measured by western blotting. **(B)** Western blot analysis of p62 and LC3 in A549/DDP cells incubated with autophagy inhibitor HCQ.

FIGURE S5 **(A)** Cells were treated by cisplatin with or without scutellarin, the protein expressions of AKT and p-AKT were determined by western blotting. **(B)** The expression levels of LC3, AKT, p-AKT were detected by western blotting in A549/DDP cells coincubated with MK-2206. **(C)** Western blot analysis of the protein of *c-met*. **(D)** The effect of crizotinib on the expression of LC3, AKT, p-AKT, c-met was analyzed by western blot analysis.

FIGURE S6 **(A)** The expression levels of LC3, p62, p53, p-ERK1/2, p-AKT, c-met in mice tumor were detected by western blot analysis.
