# Supplementary figures and images for: Scutellarin Increases Cisplatin-Induced Apoptosis and Autophagy to Overcome Cisplatin Resistance in Non-small Cell Lung Cancer via ERK/p53 and c-met/AKT Signaling Pathways
Source: Front Pharmacol. 2018 Feb 13;9:92. doi: 10.3389/fphar.2018.00092 (PMC5816782; doi:10.3389/fphar.2018.00092)

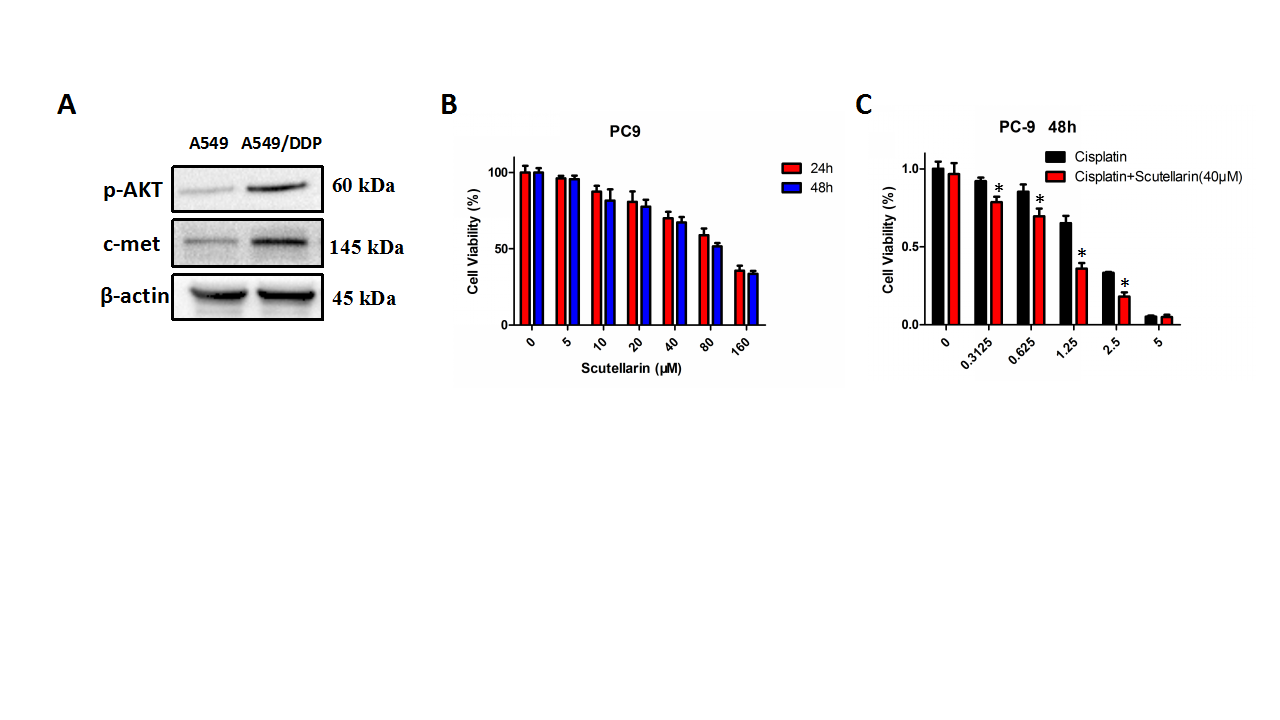

Supplement: FIGURE S1 — (A) The expression levels of c-met, p-AKT in A549 and A549/DDP cells were detected by western blot analysis. (B) PC-9 cells were treated with different concentrations of scutellarin, cell viability was measured by the MTT assay. (C) PC-9 cells were treated with cisplatin combined with scutellarin (40 μM), cell viability was measured by the MTT assay. ∗p < 0.05. [file Image_1.tif]

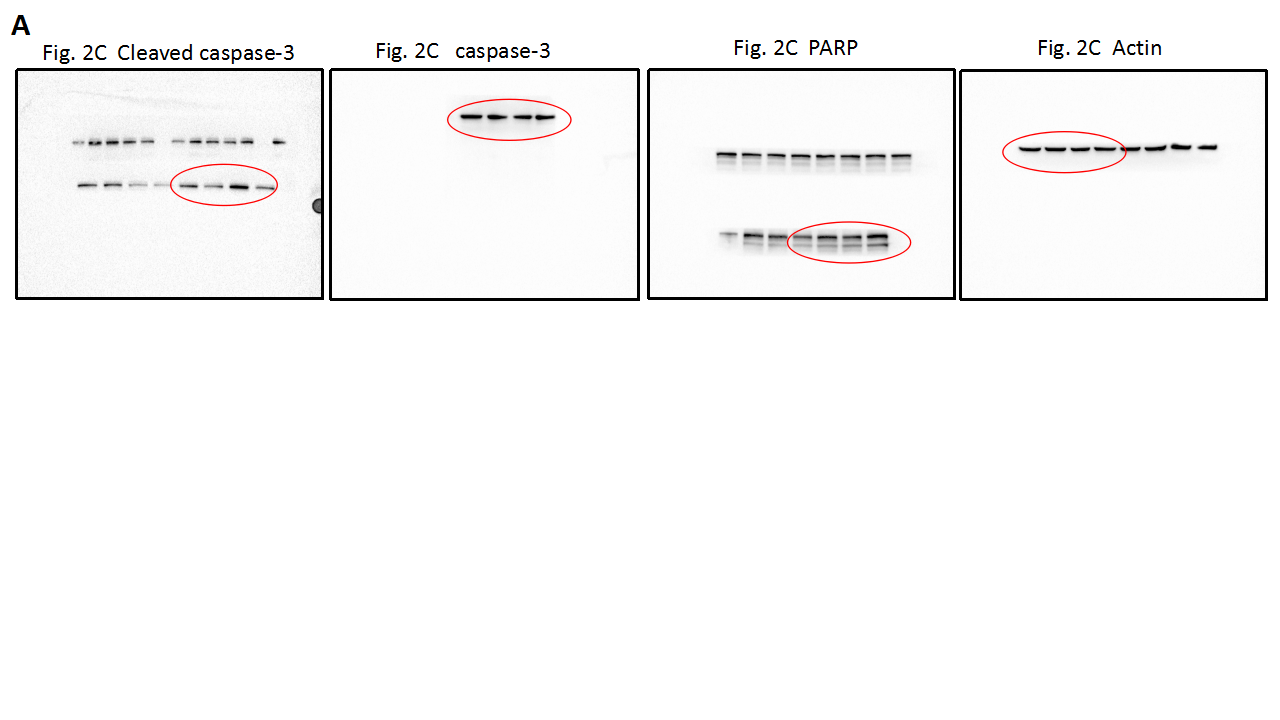

Supplement: FIGURE S2 — (A) Western blot analysis showing caspase-3, cleaved caspase-3 and PARP expression levels in A549/DDP cells treated with cisplatin, or scutellarin, or the combination. Actin was used as loading control. [file Image_2.tif]

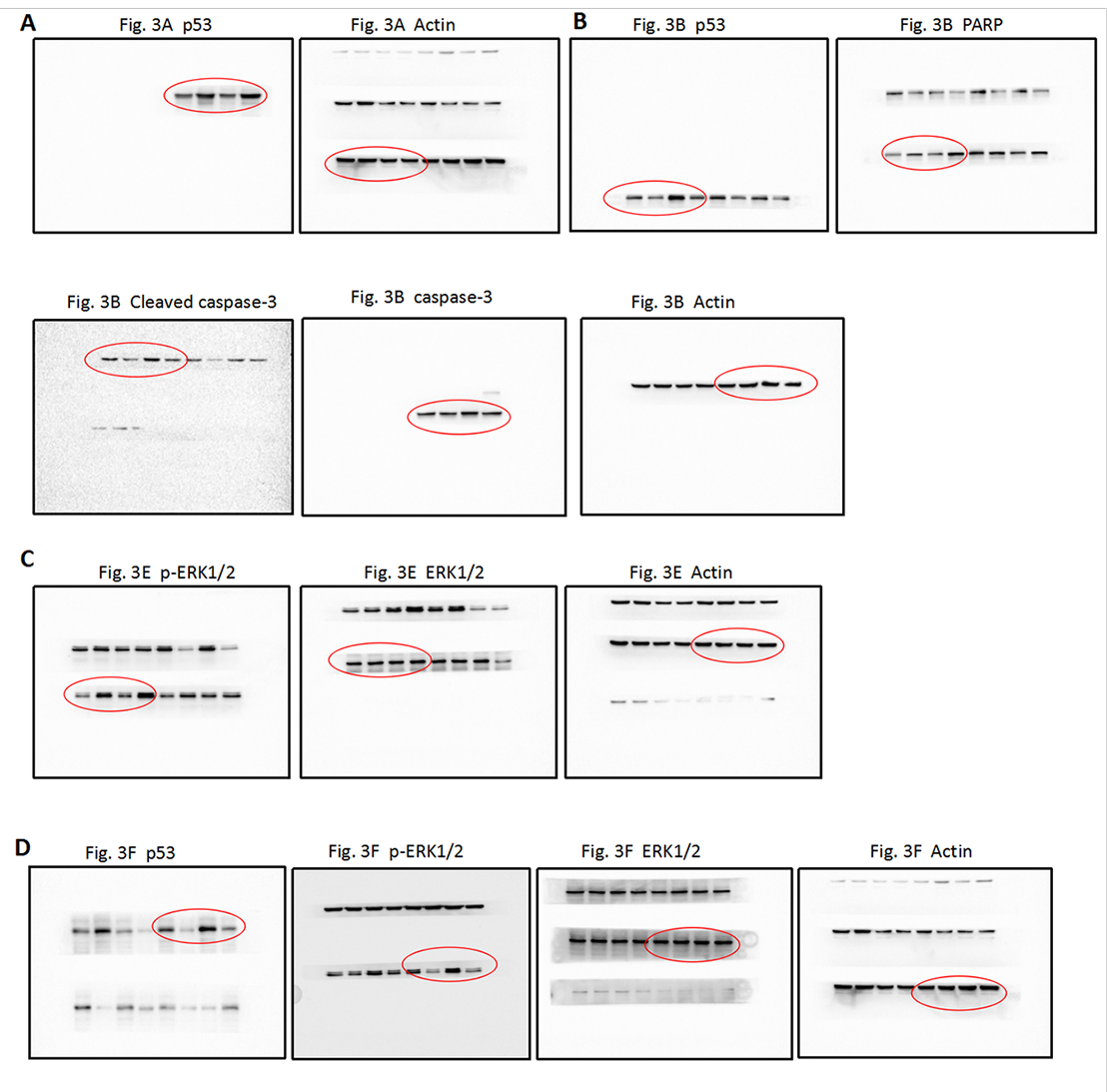

Supplement: FIGURE S3 — (A) Western blot analysis of p53 protein in A549/DDP cells treated by cisplatin, or scutellarin, or the combination. (B) The effect of p53 inhibitor PFT on p53, caspase-3, cleaved caspase-3 and PARP expression levels were measured by western blot analysis. (C) The expression of p-ERK1/2, ERK1/2 were determined by western blot analysis. (D) The level of p-ERK1/2, ERK1/2, p53 in A549/DDP cells were measured in the presence of ERK1/2 specific inhibitor U0126. [file Image_3.tif]

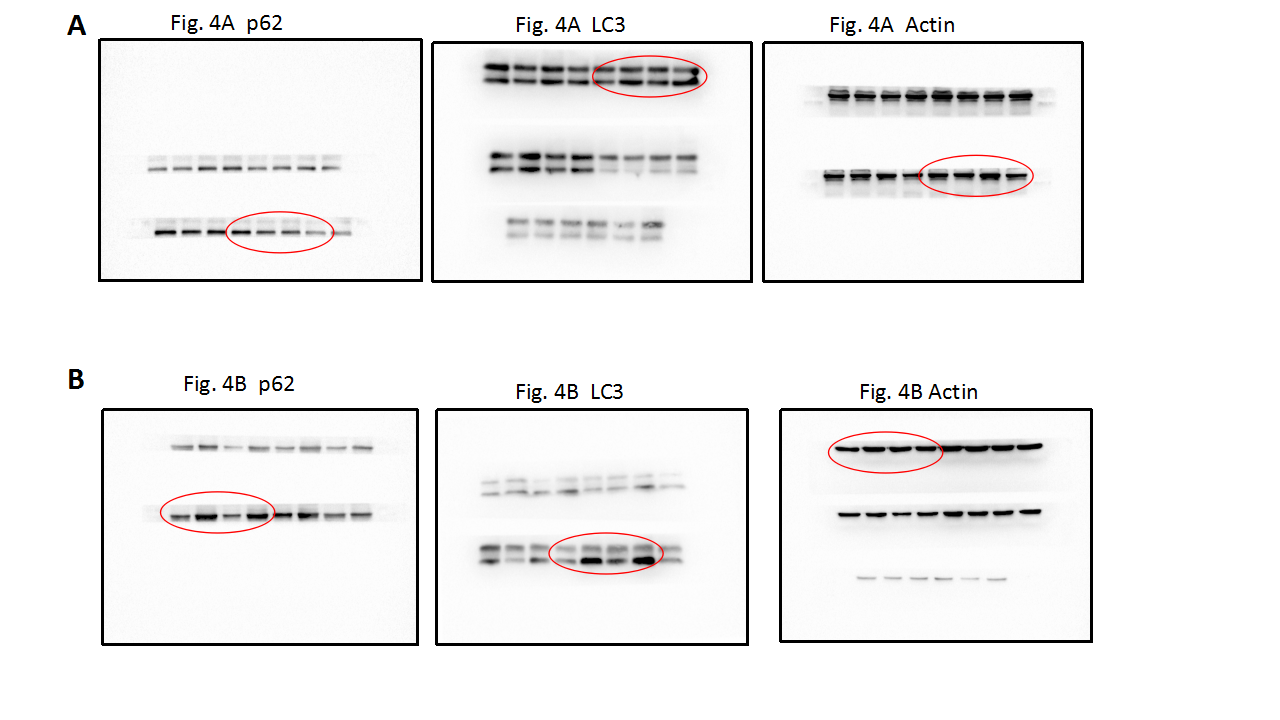

Supplement: FIGURE S4 — (A) A549/DDP cells were treated with cisplatin or scutellarin, or the combination for 48 h, the expression of p62 and conversion of LC3-I to LC3-II were measured by western blotting. (B) Western blot analysis of p62 and LC3 in A549/DDP cells incubated with autophagy inhibitor HCQ. [file Image_4.tif]

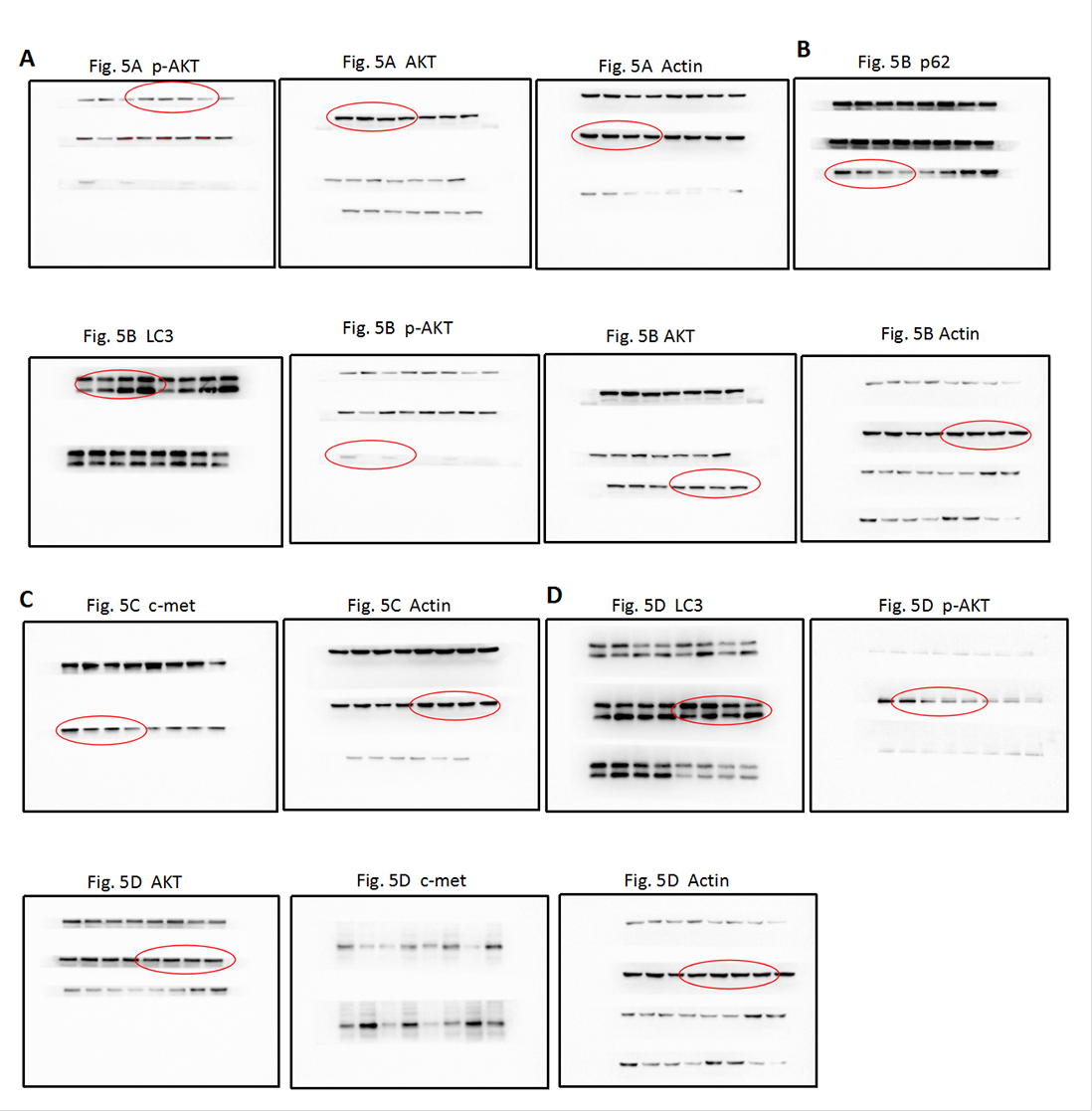

Supplement: FIGURE S5 — (A) Cells were treated by cisplatin with or without scutellarin, the protein expressions of AKT and p-AKT were determined by western blotting. (B) The expression levels of LC3, AKT, p-AKT were detected by western blotting in A549/DDP cells coincubated with MK-2206. (C) Western blot analysis of the protein of c-met. (D) The effect of crizotinib on the expression of LC3, AKT, p-AKT, c-met was analyzed by western blot analysis. [file Image_5.tif]

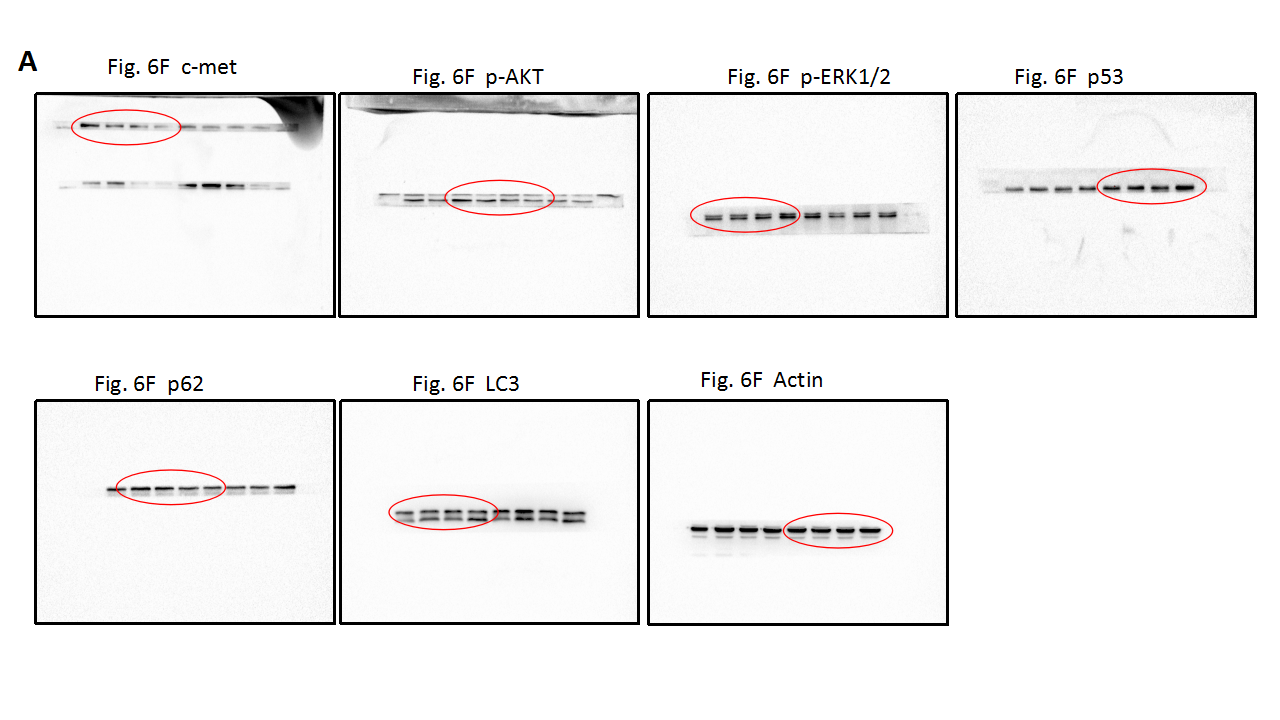

Supplement: FIGURE S6 — (A) The expression levels of LC3, p62, p53, p-ERK1/2, p-AKT, c-met in mice tumor were detected by western blot analysis. [file Image_6.tif]
